# Oscillatory IL-2 stimulus reveals pertinent signaling timescales of T cell responsiveness

**DOI:** 10.1371/journal.pone.0203759

**Published:** 2018-09-18

**Authors:** Linda E. Kippner, Melissa L. Kemp

**Affiliations:** The Wallace H. Coulter Department of Biomedical Engineering, Georgia Institute of Technology and Emory University, Atlanta, Georgia, United States of America; Bioinformatics Institute, SINGAPORE

## Abstract

Cell response to extracellular ligand is affected not only by ligand availability, but also by pre-existing cell-to-cell variability that enables a range of responses within a cell population. We developed a computational model that incorporates cell heterogeneity in order to investigate Jurkat T cell response to time dependent extracellular IL-2 stimulation. Our model predicted preferred timing of IL-2 oscillatory input for maximizing downstream intracellular STAT5 nuclear translocation. The modeled cytokine exposure was replicated experimentally through the use of a microfluidic platform that enabled the parallelized capture of dynamic single cell response to precisely delivered pulses of IL-2 stimulus. The *in vitro* results demonstrate that single cell response profiles vary with pulsatile IL-2 input at pre-equilibrium levels. These observations confirmed our model predictions that Jurkat cells have a preferred range of extracellular IL-2 fluctuations, in which downstream response is rapidly initiated. Further investigation into this filtering behavior could increase our understanding of how pre-existing cellular states within immune cell populations enable a systems response within a preferred range of ligand fluctuations, and whether the observed cytokine range corresponds to *in vivo* conditions.

## Introduction

The cytokine Interleukin-2 (IL-2) is an essential part of a functional immune system, playing a vital part in promoting tolerance and immunity. Its main role is through a with wide ranging impact on the function of immune cells, most notably on T cells, both as a growth factor [1] and as a regulator of T cell immune function [2, 3].

The IL-2 receptor (IL-2R) is comprised of three polypeptide subunits, α, β,and γ [4, 5]. Individually, the three subunits bind IL-2 with low to intermediate affinity [6] [7, 8], but upon the stepwise formation of a heterotrimeric receptor complex, their combined properties enable efficient ligand capture and subsequent cell response [6, 9–14]. While the IL-2 specific α subunit contributes the strongest affinity for the ligand but lacks a cytosolic component, the β and γ subunits are shared with other cytokine signaling pathways and contain membrane-spanning domains to allow for the initiation of an intracellular signaling transduction in response to ligand binding. Receptor-ligand interaction results in activation of cytosolic protein tyrosine kinases (PTK), such as members of the janus tyrosine kinase (JAK) family [15, 16]. In Jurkat cells, JAK1 and JAK3 associate with receptor subunits β and γ, and initialize a signaling cascade. Downstream of JAK, phosphorylation of cytosolic STAT5 allows for its dimerization and import into the nucleus [17–19], where it operates as a transcription factor. The three subunits of the IL-2 receptor are all expressed in varying numbers among cells of a population [20, 21]; thus, the number of trimeric receptors available to capture extracellular IL-2 and transduce signal will differ between individual cells, which in turn will lead to varying behavior in cell response. Consequently, it is to be expected that a population average will not be sufficient to capture the range of responses in a cell population.

Sensitivity of cellular response to quick oscillations of input is observed in other systems, such as intracellular T cell Ca^2+^ dynamics in response to extracellular H_2_O_2_ oscillations [22]. This raises the question of how such dynamics could affect cellular response to natural ligands that undergo binding and internalization such as cytokines. We investigated whether T cells respond differently to rapid IL-2 fluctuations of varying length, a feature that would allow the cell population a more fine-tuned response to extracellular stimulus such as preferential ranges of temporal ligand dynamics. Cell signaling systems often respond to extracellular ligand with exquisite sensitivity to minute changes in concentration. Pre-equilibrium sensing and signaling (PRESS) could occur in a system where the downstream response is faster than the time needed to reach equilibrium for receptor-ligand interaction at the cell surface, allowing the cells to distinguish between pre-equilibrium doses of ligand [23]. PRESS has been demonstrated to expand and shift the dynamic range of input ligand concentrations for orientation/polarization in chemotactic gradients. Based on the kinetics of its receptor-ligand interaction and the downstream processes, the IL-2 ligand-trimeric receptor system was recently proposed to be regulated by pre-equilibrium sensing and signaling [23]. The microfluidic delivery of time-varying IL-2 enable our investigation of the effects of rapidly fluctuating extracellular IL-2 levels on T cell response by subjecting cells to input in a regime that is below equilibrium levels and at physiologically relevant concentrations.

Clinical side effects caused by simultaneous stimulation and suppression of immune function as well as systemic toxicity have limited the therapeutic use of IL-2. One recent approach to prevent such undesired effects is through the engineering of IL-2 receptors [24]. Alternatively, refining delivery dynamics of recombinant IL-2 may yield higher cellular responses to the cytokine at lower doses. In our model system, we remove de novo synthesis of IL-2Rα and subsequent IL-2 production [25] as the limiting factor by using Jurkat cells which constitutively produce IL-2, such that the cells are primed with all three subunits present at the cell surface. Under these conditions, we explore the rate limiting steps of receptor complex formation and investigate kinetics and expression levels of the IL-2R subunits critical for signaling response under clinically relevant doses of oscillatory IL-2 conditions.

## Methods

### Cell culture and transfection

Jurkat cells and Human Embryonic Kidney (HEK) 293T cell lines were purchased from ATCC and maintained in RPMI-1640 medium without phenol red (Lonza), supplemented with 10% FBS (Sigma), L-glutamine (Fisher Scientific), 1% MEM non-essential amino acids (Mediatech), 10 mM HEPES (Mediatech), 1 mM sodium pyruvate (Mediatech), 50 U/mL penicillin and 50 μg/mL streptomycin (Fisher Scientific) at 37°C 5% CO_2_. For transfection of plasmid containing GFP-labeled STAT5 (Origene Technologies), HEK 293T cells were transfected using the Neon transfection system (Thermo Fisher). After two days of culture following transfection, supernatant containing retrovirus was collected and used to transfect Jurkat cells by spinoculation. Following spinoculation and recovery, GFP-positive Jurkat cells were collected from the population by sorting using a BD FacsAria Fusion cell sorter. STAT5-GFP expressing Jurkat cells were maintained in culture as above.

### Computational model

The model simulates cellular response to periodic cytokine stimulus, using Jurkat T cells as the model cell and IL-2 as the cytokine. The initial framework for our model was a previously published model of cellular proliferation in response to IL-2 [26]. Our model was constructed in the MatLab Simbiology platform and run using a stiff ODE15 solver. Values for all model rates are shown in Table 1 and model equations are provided in Table A of S1 File.

**Table 1:** Parameter values for computational model. Process numbers correspond to numbers in model overview in Fig 1.

Parameter	Process	Definition	Value	Units	Source
kon1	1	Association of IL2Rα and IL-2 at cell membrane	1.4E+07	M^-1^s^-1^	Feinerman 2010 [20]
koff1	2	Dissociation of IL2Rα-IL2 at cell membrane	0.4	s^-1^	Feinerman 2010 [20]
kon2	3	Association rate for IL2Rα-IL2 to IL-2Rβ / IL-2Rγ	3.6E-04	M^-1^s^-1^	Fitted to model output by genetic algorithm
kr	4	Cell surface dissociation of receptor and ligand	2.3E-04	s^-1^	Wang 1987 [9]
ke	5	Internalization of receptor-ligand complex	6.67E-04	M^-1^s^-1^	Fallon 2000 [26]
kre	6	Intracellular dissociation of ligand and receptor	0.00184	s^-1^	Fallon 2000 [26]
kfe	7	Intracellular association of ligand and receptor	1.84E-06	M^-1^s^-1^	Fallon 2000 [26]
kh	8	Degradation of internalized receptor and ligand	5.83E-04	M^-1^s^-1^	Duprez 1988 [27]
kx	9	Recycling	0.00175	M^-1^s^-1^	Ghosh 1994 [28]
kt	10	Constitutive internalization of unbound receptor	1.17E-04	s^-1^	Hemar 1995 [29]
Vs	11	Constitutive receptor subunit synthesis rate	0.25	M·s^-1^	Wiley 1981 [30]
ksyn	12	Enhanced receptor synthesis due to receptor-ligand interaction	1.83E-05	s^-1^	Smith 1985 [31]
ku1	13	STAT5 translocation delay steepness constant	2.5E-03		Fitted to experimental data
ti	13	STAT5 translocation delay inflection constant	2.0E+03		Fitted to experimental data
b	13	STAT5 translocation delay scaling constant	3.5E-03		Fitted to experimental data
d	13	STAT5 translocation delay constant	1.80		Fitted to experimental data
NA		Avogadro's constant in picoscale	6.02E+11	Mol^-1^	
Ve		Total endosomal volume	1.0E-14	L	French 1995 [32]
Vol		Volume of microfluidic system	2.01E-07	L	Chingozha 2014 [33]

**Fig 1:**
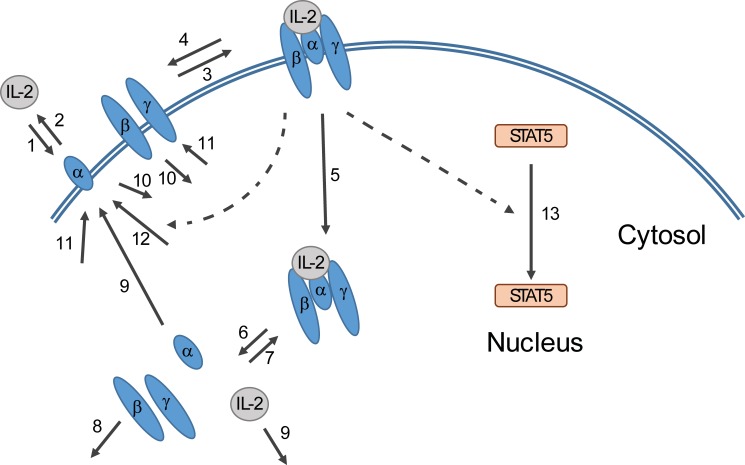
Overview of computational model. The model incorporates the formation of receptor-ligand complex from extracellular IL-2 and receptor subunits at the cell membrane (1–4). The complex is internalized (5) and its components sorted for intracellular degradation (8) or recycling (9). The binding of IL-2 by its receptor initiates a downstream signaling cascade that results in translocation of STAT5 from the cytosol to the nucleus (13). This is represented by a delay function affected by the number of receptor-ligand complexes present at the cell membrane. The model also incorporates constitutive (11) and IL-2 induced (12) production of receptor subunits and consecutive internalization of unbound receptor (10). Dashed arrows indicate an effect of a species on a reaction. Numbers by reaction arrows correspond to process numbers in Table 1.

Fig 1 shows an overview of the species and reactions included in the model, which was adapted to incorporate subunit-level detail of the IL-2 receptor, as well as downstream translocation of STAT5 from the cytosol to the nucleus. The volume of the model was customized to our experimental microfluidics setup rather than a bulk cell culture system, such that the effective extracellular concentration of IL-2 was reflected in the model simulations. The time scale was likewise altered to reflect our short (≤ 1 hour) timescale of interest. Transcriptional processes, both constitutive and IL-2 induced, were included in the model; however, the longer time scale needed for these processes meant that their impact on the model outcome was minimal. The initial steps of the model describe binding of IL-2 by the three IL-2 receptor subunits at the cell surface, and the sequential assembly of the ligand-receptor complex. This is followed by the resulting downstream cellular response to signaling by the ligand-receptor complex in the form of STAT5 translocation from the cytoplasm to the nucleus. In our model, the steps between receptor-ligand interaction to STAT5 translocation are represented in a simplified fashion by a logistic delay function, where translocation of STAT5 is dependent on the number of receptor-ligand complexes present at the cell surface.

IL-2 is bound to the three subunits of the receptor by stepwise assembly with IL-2α strengthening the bond to the ligand allowing first IL-2β and then IL-2γ to bind to the complex [11]. This order represents the canonical pathway for IL-2 receptor-ligand complex formation. While higher expression and higher affinity of IL-2α makes the canonical pathway likely, it is theoretically possible that other assembly combinations could occur, depending on cell characteristics such as the relative numbers of available subunits. These other pathways could then have an effect on cellular response to IL-2, in particular if IL-2β is expressed in excess of IL-2α [34]. Our model assumes an excess of IL-2α over IL-β, and does not incorporate alternative binding pathways.

This step-wise formation of the receptor-ligand complex is represented by two steps in our model, with the initial step being the capture of IL2 by IL-2Rα and with the subsequent addition of IL2β and IL-2Rγ simplified into one step. The IL-2Rβ and IL-2Rγ subunits of the receptor were modeled as one combined species, under the assumption that they were co-localized prior to complex formation. According to Feinerman et al, [20], adding the association of first IL-2Rβ, then IL-2Rγ did not result in model outcome different from that of adding the two subunits simultaneously. We thus considered this simplification justified. Likewise, as experiments have reported no presence of alternate receptor dimer combinations (IL-2Rα/IL-2Rγ or IL-2Rβ/IL-2Rγ) in the absence of IL-2, we disregarded this possibility of subunit competition when constructing our model [35]. Initial values for receptor subunits were based on randomized sampling from single cell flow cytometry data as described in [36], here measuring the expression levels of IL-2Rα and IL-2Rβ on Jurkat T cells and converted to quantified subunit numbers within ranges reported in literature [20, 29, 37, 38]. Upon formation of the receptor-ligand complex at the cell membrane, the complex is internalized and the components undergo differential sorting [29]. The model incorporates this internalization of the ligand-receptor complex, as well as degradation of the IL2Rβ and IL2Rγ subunits and recycling of the IL2Rα subunit and the ligand [27, 29]. In order to mimic our experimental conditions which occurred under constant flow, recycled IL-2 was modeled as lost from the model system and not available to the cell. Downstream steps from surface complex formation to STAT5 translocation were described in a simplified fashion as a time delay function where the translocation of STAT5 was defined as dependent on the number of receptor-ligand complexes present on the cell surface. The rate of the delay was determined by fitting to experimentally obtained data from responding Jurkat cells after constant stimulation with 100 pM IL-2 (Figure A in S1 File and a single cell example in S1 Movie).

IL-2 concentrations of 10pm and 100pM were tested in the model, with 10 pM being the canonical threshold for triggering T cell response [39]. In a clinical setting, concentrations between 1 pM and 100 pM have been reported as therapeutically relevant [40], while concentrations above 100 pM induce undesirable inflammatory responses [41, 42]. With this in mind, we considered a range of 10 pM to 100 pM to be biologically relevant and these two values were tested in our model.

We investigated model response to pulsatile input of IL-2 using custom MatLab code, which delivered a pre-defined set of IL-2 pulses interspersed by recovery pauses to the modeled system. IL-2 was delivered at pulse lengths of 0.5, 1, 2, 3, 4, or 5 minutes followed by pause lengths of the same durations, for a total of 36 combinations of pulse and pause lengths. Total simulation time was set to one hour for all IL-2 input settings. Two dynamic model outputs were collected: the total number of receptor-ligand complexes present at the cell membrane (R-L), and the nuclear localization of STAT5. Under the assumption that only complexes of trimeric IL-2 receptor and bound IL-2 present at the cell membrane are able to initiate downstream signaling, the R-L value was used as a first indicator of cell response to IL-2. The translocation of STAT5 from cytosol to nucleus is a direct downstream effect of IL-2-receptor interaction and was represented in the model as the ratio of nuclear to cytosolic STAT5 (Sr). This was used as a downstream indicator of cellular response to IL-2.

### Cell response assay in microfluidic devices

Two layer microfluidic devices were fabricated as previously described [43]. Briefly, the devices were molded from 10% polydimethylsiloxane (PDMS), assembled, and plasma bonded to glass slides to enable imaging. The two-layer design of the device allowed for immobilization of suspension cells by horizontal flow in one layer and for delivery of stimulus to all cells in the trap simultaneously by perpendicular flow from the top layer. Before loading onto the device, STAT5-GFP Jurkat cells were resuspended in HBSS with 0.5% FBS and incubated with Hoechst 33258 nucleic acid dye (Sigma) and Wheat Germ Agglutinin (WGA) Alexa Fluor 647 membrane stain (Fisher Scientific) for 10 minutes at 37°C. Following incubation, cells were washed, resuspended in growth medium, and loaded into the device using gravity flow. After loading, device inlet tubing for the two inlets was connected to growth medium with and without IL-2, and delivery was controlled using a custom-built pressure box with inlet pressure set to 1 psi. A custom GUI controlled pinch valves to enable precise delivery of stimulus and medium to the trapped cells. Prior studies with fluorescein labeled buffer have characterized that the pulsatile properties of this platform are well-matched to the desired input function [22]. Cells were exposed to either a constant flow of medium containing 10 pM or 100 pM IL-2 or to a pulsatile input of 100 pM IL-2, alternating with growth medium without IL-2. Model predictions informed the choice of four different settings for pulse and recovery times. Time-lapse images were taken every 60 seconds in the GFP and DAPI ranges at 20x magnification using a PerkinElmer UltraVIEW VoX spinning disk confocal microscope with a Nikon Ti-E camera. WGA labeling of the cell membrane facilitated the identification of cells during imaging setup in order to minimize photobleaching of GFP. Total experimental time was set to one hour to minimize possible effects of IL-2 mediated gene upregulation [44].

### Quantification of STAT5 translocation

Images taken during cell stimulation were analyzed using custom MatLab code. In order to distinguish between the whole cell and the nuclear compartment, masks were created to capture areas of interest (cells) for each image field and to create areas of interest (cytosol and nucleus) for each cell. Local background GFP intensity was subtracted from each individual whole cell and its nucleus at each time point and the ratio of GFP intensity for nuclear versus cytosolic area was then calculated for each cell at each time point. For time points where image quality was deemed too low, data was interpolated by the mean of the two time points immediately preceding and following for the individual cell. The resulting STAT5-GFP ratio data generated dynamic single-cell traces of STAT5-GFP translocation. For each treatment group, the time series data for all cells were analyzed by hierarchical clustering using Ward’s minimum variance method with Euclidean distance (Figure C in S1 File). Based on the clustering results, we set a STAT5-GFP ratio threshold to define responding cells of all response patterns versus non-responding cells. Where the normalized ratio of nuclear to cytosolic GFP reached 1.5 or higher for two or more time points during the one hour time course, the cells were categorized as responders to IL-2 stimulus (Fig 6A top, 6B top, 6C top, and 6D top). The mean of the maximum ratio of nuclear to cytosolic STAT5-GFP, as well as the mean area under the curve for the same ratio were tested using one-way ANOVA with *post hoc* Tukey test for responding versus non-responding cells from all 4 fluctuating IL-2 input settings.

### Comparison of STAT5 and STAT5-GFP in transfected Jurkat cells

Cytoplasmic and nuclear lysates were obtained from transfected and sorted STAT5-GFP expressing cells. A Western blot was run using 20 μg of total protein sample from each fraction on a 10% SDS-PAGE gel followed by transfer to a PVDF membrane. The membrane was blocked with Near Infra-Red Blocking Buffer (Rockland Immunochemicals) and probed using anti-STAT5 antibody (BioLegend) at 1:1000 dilution in 4°C overnight, followed by secondary anti-mouse antibody (IR dye 680CW donkey anti-mouse, Licor Biosciences) at 1:10,000 for 1 hour at room temperature. The membrane was imaged using a Licor Odyssey CLx Imaging System and analyzed using Image Studio software to quantify the relative intensities of the bands for STAT5 and STAT5-GFP in the two cell compartments.

### Relative levels of IL-2Rα and IL-2Rβ within the Jurkat population

In order to investigate relative levels of IL-2Rβ within the Jurkat cell population, cells were incubated with antibody against IL-2Rβ. 100,000 cells were resuspended in fresh medium and incubated with primary antibody against IL-2Rβ (Novus Biologicals) at 1:10 dilution for one hour at room temperature. The cells were then washed three times and incubated for one hour with Alexa 647-labeled secondary antibody (Life Technologies) at 1:200 dilution for one hour at room temperature, followed by three additional washes. Cells were analyzed in two ways: by flow cytometry using a BD LSR Fortessa and by fluorescent microscopy using a PerkinElmer UltraVIEW VoX spinning disk confocal microscope with a Nikon Ti-E camera. The relative intensity of the Alexa 647 signal was quantified for each cell and used to assess the heterogeneity of available IL-2Rβ within the population. In order to investigate relative levels of IL-2Rα and IL-2Rβ on the same cell, cells were incubated with FITC-labeled antibody against IL-2Rα (BioLegend) and APC-labeled antibody against IL-2Rβ. Cells were analyzed using flow cytometry, as above.

## Results

### Model simulations predict differential responses to oscillatory IL-2 input functions

**Fig 2:**
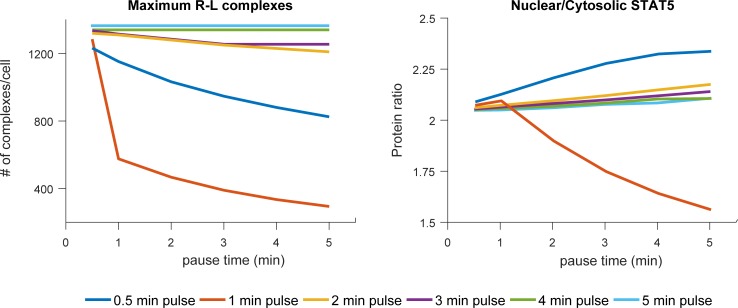
The model predicted variation in maximum response due to IL-2 pulse metrics. a) Maximum receptor-ligand complexes per cell, and b) STAT5 nuclear/cytosolic ratio per cell, during a one hour simulation under different pulsatile IL-2 input conditions. Pulse length is indicated by legend and pause length on the x axis. All simulations were run using 100 pM IL-2.

Our model describes downstream responses of T cells upon binding IL-2, modifying a computational framework first developed by Fallon & Lauffenburger [26] to include features of interest to us associated with the earliest events of IL-2 receptor ligation and to exclude longer timescale events which are beyond the scope of our experimental context. The IL-2 receptor is represented in our model as individual subunits, which combine in a stepwise fashion upon binding IL-2 to form a heterotrimeric receptor. The receptor-ligand complex is assumed to initiate an intracellular signaling pathway, resulting in the translocation of STAT5 from the cytosol to the nucleus, and this translocation is used as a model output indicating cell response. In addition to this chain of events, the internalization and subsequent recycling and degradation of the receptor-ligand complex are included in the model, as this affects the number of cell surface receptor-ligand complexes available for initiation of cell response. The steps between receptor-ligand interactions to STAT5 translocation are represented in a simplified fashion by a delay function, where translocation of STAT5 is dependent on the number of receptor-ligand complexes present at the cell surface. The model allows for tight control of ligand input, simulating addition and removal of IL-2 to the extracellular environment. We simulated a range of 36 input combinations in order to investigate cellular dynamics in response to pulsatile IL-2 stimulation at below equilibrium levels. Each simulation setting represented a one hour time course with pulses of 30 seconds to 5 minutes of IL-2 followed by recovery times (pauses) of 30 seconds to 5 minutes. The model under consideration represented an “average cell” with receptor subunit levels defined by values within the mean range reported in literature. The resulting cellular trajectories indicated a range of cellular response depending on the IL-2 input, as measured by the maximum number of receptor-ligand complexes (R-L) present at the cell membrane (Fig 2A), as well as the maximum ratio of nuclear to cytosolic STAT5 (Fig 2B). The maximum level of R-L and maximum ratio of nuclear to cytosolic STAT5 here represented the maximum cell surface and intracellular responses to IL-2 in our systemThe maximum level of cellular response varied with length of stimulus pulse and recovery time (Fig 2A and 2B). Both output metrics showed a decrease in peak value as recovery time between IL-2 pulses was increased, with the effect of recovery time being less influential as the length of stimulus pulses was increased. For both metrics, the response was most highly affected by changes in recovery time when pulse time was set to 1 minute. As pulse length decreased or increased from one minute, the change in maximum response due to altered recovery time decreased. For shorter stimulus pulses, a decrease in maximum response was predicted with longer recovery times. Again, this effect was most marked when pulse length was one minute.

### Model results show variation in maximum cell response depending on receptor subunit heterogeneity

**Fig 3:**
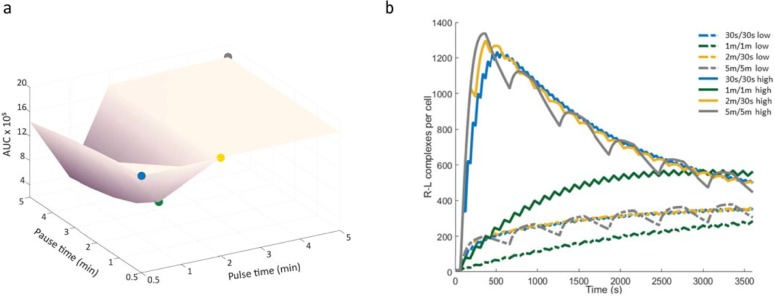
Difference in receptor-ligand complex formation due to cell-to-cell variability in IL-2Rβ/γ numbers. Model results showing a) the difference in area under the curve for receptor-ligand complexes per cell and b) the change in receptor-ligand complexes per cell over time due to variability (high and low initial expression levels) of IL-2Rβ/γ numbers. The difference in initial subunit numbers affects cell response to pulsatile IL-2 input in varying degrees across all combinations of ligand pulse and recovery times, with three of the four input settings resulting in similar response profiles. Colored boxes in a) represent stimulus (IL-2) inputs of 30 seconds/30 seconds, one minute/one minute, two minutes/30 seconds, and five minute/five minute pulse/pause combinations, corresponding to the colors and input settings used in b). IL-2 concentration was 100 pM and total simulation time one hour for all pulsatile stimulus combinations. High and low expressing cells had initial values set to 1500 and 150 IL-2Rβ/γ per cell, respectively. Solid lines in b) represent cells with high IL-2Rβ/γ and dashed lines represent IL-2Rβ/γ expression.

**Fig 4:**
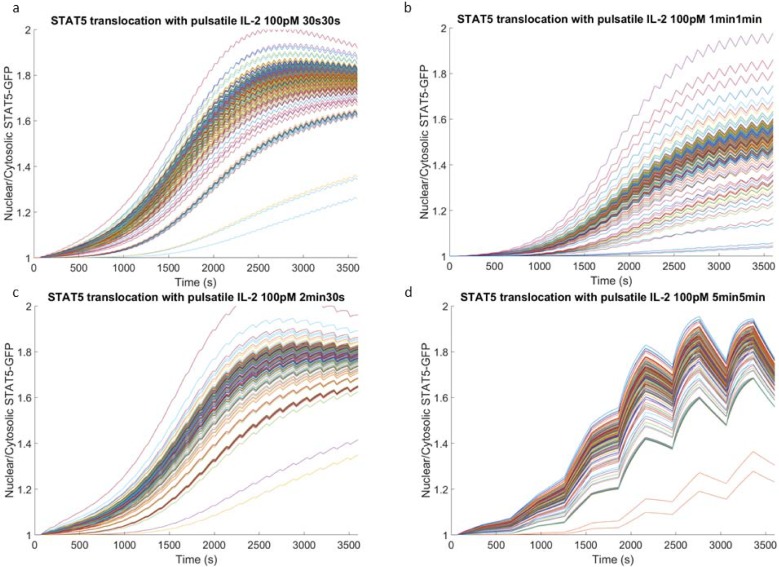
Predicted cell response to pulsatile IL-2 with IL-2Rα and IL-2Rβ/γ expression based on population sampling. Model results showing predicted population response to pulsatile input of 100pM IL-2 for a) 30 seconds/30 seconds, b) one minute/one minute, c) two minutes/30 seconds, and d) five minute/five minute pulse/pause combinations. Initial values for IL-2Rα and IL-2Rβ/γ were based on random sampling from flow cytometry data for Jurkat cells co-labeled for IL-2Rα and IL-2Rβ.

**Fig 5:**
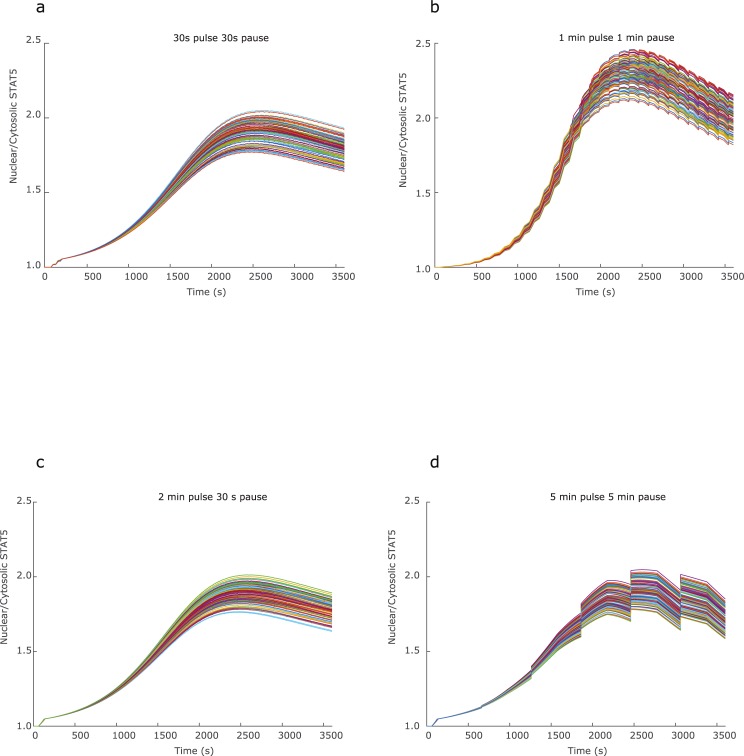
Random sampling within 10% variation in all model parameter values for the 4 pulsatile stimulus settings defined in Fig 3.

The three receptor subunits of the IL-2 receptor are present at the cell surface in numbers that vary between cells in a population. Activated T cells have been found to express the subunits of the IL-2 receptor in ranges up to two (IL-2Rβ) and three orders of magnitude (IL-2Rα) [20, 21]. Due to this heterogeneity in expression, individual cell response to IL-2 can be assumed to vary with the availability of receptor subunits. Our model addresses this population heterogeneity by allowing for simulation of high and low levels of unbound receptor subunits at the resting level. Results for cells with 1000 or 1500 IL-2β/γ indicate that the cells achieve different levels of maximum response, defined by both the maximum number of receptor-ligand complexes present at the cell membrane and the ratio of nuclear to cytosolic STAT5, to the same input of IL-2. The difference in dynamic response between simulated cells with low versus high initial levels of IL2Rβ/γ subunits showed a lower overall response level as an effect of lower IL-2β/γ subunit levels across all input combinations, as compared to cells with higher subunit levels (Fig 3A). As the IL-2 concentration used was chosen to avoid ligand saturation, cell response dynamics under constant ligand input could be expected to be similar for cells with different initial numbers of available receptor. Under these conditions, the main expected difference would be how rapidly cells respond and how sustained this response can be. This can be expected to be of importance in a system where there is rapid change in ligand availability, such as under fluctuating ligand input conditions. This was especially apparent for the input settings where the pulse time for extracellular IL-2 availability was 1 minute and the recovery time between pulses was varied. In this input range, cells showed the highest level of sensitivity to cell-cell variability in subunit levels, with a seven-fold difference in maximum response and a four-fold difference in area under the curve as recovery pulse time increased from 30 seconds to five minutes (Fig 3A, Figure D in S1 File). Likewise, response dynamics over a one hour simulation time showed a range of response profiles, with cells responding to one minute IL-2 pulses and one minute recovery pauses showing a response profile that differed from the other input settings (Fig 3B). We further tested the effect of subunit heterogeneity by allowing our model to set expression levels from random sampling of a probability density function of receptor levels generated from flow cytometry data. For each randomly sampled cell, the corresponding expression levels of IL-2Rα and IL-2Rβ were quantified to values within ranges reported in literature and used as initial values for the corresponding model species. Model populations of 200 cells were simulated using four pulsatile 100pM IL-2 inputs. The resulting ensemble of cell responses (Fig 4) showed the broadest range of behavior within the cell population for the 1min/1min pulsatile IL-2 input combination. In addition to receptor subunit numbers, cell heterogeneity was also explored in the context of reaction rates. This was done through the parameter values in our model, which were randomly varied within a 10% range above and below original parameter values. The predicted range of STAT5 nuclear translocation was recorded for 100 variations of the model under four pulsatile IL-2 input settings (Fig 5). The model predictions showed the highest ratio of nuclear to cytosolic STAT5 when IL-2 was delivered in one minute pulses with one minute pauses (Fig 5B). The range of STAT5 ratio achieved at the end of the one hour simulation time was likewise broadest for this pulsatile setting (Table 2).

**Table 2:** Responding cells and average nuclear/cytosolic STAT5-GFP.

Condition	30s/30s	1m/1m	2m/30s	5m/5m
Responding cells (%)	30	**51**	37	39.6
Average max norm STAT5 ratio responders	2.7 ± 0.76	**4.34 ± 4.58**	2.1 ± 1.40	2.3 ± 0.88
AUC non-responders	56.2 ± 9.8	**54 ± 11.5**	58.7 ± 11.9	58.0 ± 12.6
AUC responders	98.5 ± 13.2	**135.5 ± 125.2**	71.8 ± 32.0	81.1 ± 21.5

### Jurkat cells in culture are pre-primed to express IL-2Rα

Jurkat cell constitutively produce STAT5, which means that tracking introduced GFP-labeled STAT5 does not reflect the total STAT5 in the system due to the presence of unlabeled endogenous protein. In order to determine the proportion of GFP-labeled STAT5 in the transfected cells, lysates of the cytosolic and nuclear fractions from transfected and sorted cells were analyzed by Western blot. The quantified results showed that the mean ratio of cytosolic GFP-labeled STAT5 to endogenous STAT5 in the population was 0.14 (Figure B in S1 File). The level of GFP-labeled STAT5 in the nuclear fraction was below the level of detection by Western blot, but endogenous nuclear STAT5 was present at a ratio of 0.017 to endogenous cytosolic STAT5. In addition, individual cell images of GFP-labeled STAT5 indicated its presence in both cytosol and nucleus of cells prior to stimulation, indicating that some STAT5 translocation was occurring in unstimulated cells. Thus, we could consider these cells to be “primed” by IL-2 and consequently expressing IL-2Rα at the cell surface, enabling them to respond to IL-2 without the need for experimentally induced IL-2Rα upregulation.

In addition, the relative numbers of available receptor subunit IL2-Rα were compared using flow cytometry and fluorescence imaging. We observed a range of subunit expression levels within the Jurkat cell population indicating cell-cell variability spanning three orders of magnitude in the numbers of receptor subunits available for IL-2 interaction.

### Responses to clamped IL-2 stimulation reflect cellular variability within the Jurkat population

Given the numerous combinations of pulses and recovery times possible to explore experimentally, we used the model simulations to guide our selection of stimulatory conditions. To first explore how constant IL-2 stimulation (i.e. no recovery) would yield population-level responses, we subjected trapped cells to a steady flow of 10 pM and 100 pM IL-2, concentrations that the model indicated would yield below-saturation receptor-ligand complex formation at the cell surface (model results not shown). Cells subjected to 10 pM of IL-2 did not respond with any quantifiable translocation of STAT5-GFP *in vitro* (results not shown) under the time scale of interest (60 minutes) and further experiments were thus conducted using 100 pM IL-2. When exposed to a constant input of 100 pM IL-2, 33% of cells translocated STAT5-GFP to the nucleus during the one hour course of the experiment, as indicated by increased ratio of nuclear to cytosolic GFP (Figure A in S1 File). These results highlight the importance of studying single cell response rather than relying on population average.

### Preferential timescale of one minute on/one minute off input as revealed by increased number of responsive cells and increased variance

**Fig 6:**
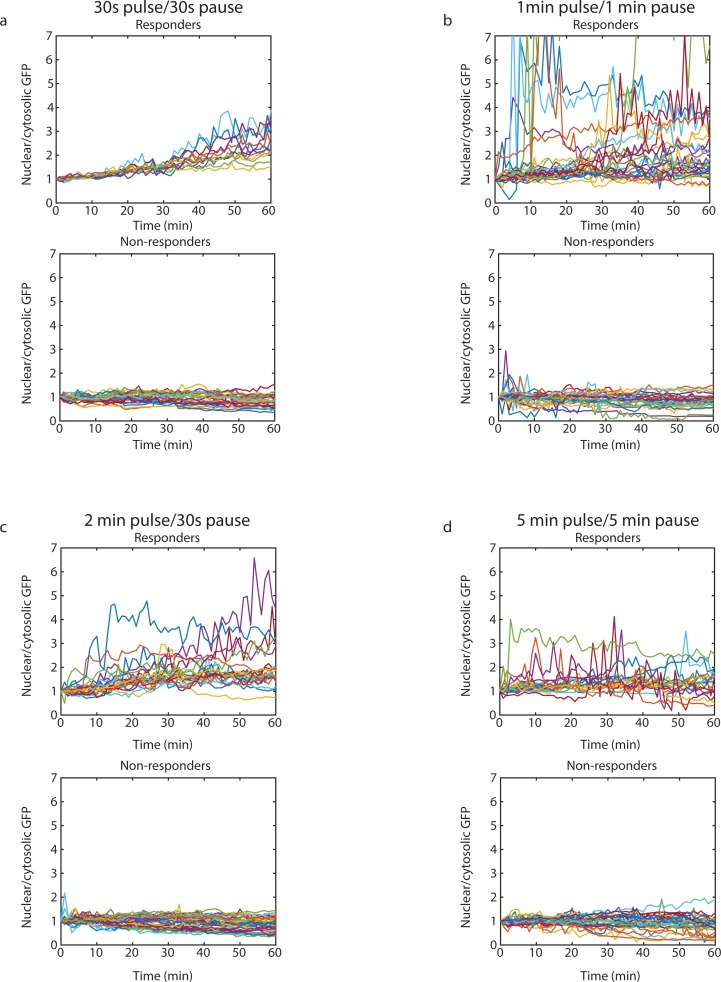
Nuclear translocation of STAT5-GFP in live cells upon oscillatory IL-2 stimulation. Nuclear translocation of STAT5-GFP in responding and non-responding Jurkat cells exposed to pulsatile input of 100 pM IL-2 with a) 30 seconds pulses and 30 seconds recovery pauses, b) one minute pulses and one minute recovery pauses, c) two minute pulses and 30 s recovery pauses, and d) five minute pulses and five minute recovery pauses. Responding cells show an increased ratio of nuclear to cytosolic GFP over to course of one hour.

In order to investigate live cell response to pulsatile IL-2 inputs, Jurkat cells were individually trapped in a microfluidic device capable of delivering tightly controlled stimulus to all trapped cells simultaneously [33]. Within the device, buffer solution containing IL-2 was delivered by orthogonal flow to cells resting in hydrodynamic traps in a near square-wave fashion. Cells were thus exposed to time-varying IL-2 inputs, and STAT5-GFP translocation was tracked over the course of an hour using fluorescent imaging. Pulsatile input settings were informed by the modeled response to cross-flow inputs of varying lengths. As our model predicted the one minute/one minute range to be of interest, our experimental tests were done at four stimulus input settings in and around this range. Of the modeled responses, pulse and recovery time combinations of 30 seconds/30 seconds, one minute/one minute, two minutes/30 seconds, and five minutes/five minutes were experimentally tested for the same concentration of IL-2. The model predicted similar maximum response for three of these four combinations as indicated by the number of receptor-ligand complexes present at the cell membrane but also predicted differences in population variability (Fig 3), leading us to investigate whether the dynamic response of the cells would yield differences in heterogeneity. After exposing Jurkat cells to the same pulsatile IL-2 stimulus *in vitro*, the resulting cell response, as indicated by STAT5-GFP translocation to the nucleus, showed distinct profiles. We observed that cells exposed to IL-2 pulses of intermediate length (one and two minutes) followed by short recovery times (one minute and 30 seconds, respectively) (Fig 6B and 6C) responded more quickly and with greater variance in response profiles than either cells exposed to short (30 seconds) pulses with short recovery (Fig 6A) or cells exposed to long (five minute) pulses and long recovery times (Fig 6D). In addition, cells exposed to one minute pulses and one minute pauses showed a higher proportion of responding cells (51%) than the other pulsatile combinations (30–40%) (Table 2). Statistical significance was found between the mean maximum ratio of nuclear to cytosolic STAT5-GFP for the responding cells between the four IL-2 input groups using one-way ANOVA (p = 0 .027), but no pairwise significant difference was found with *post hoc* Tukey test. The means of the AUC for the responding cells were statistically significant using a one-way ANOVA (p = 0 .032), with significant difference of the mean seen with *post hoc* Tukey test between the 1 minute pulse 1 minute pause group and the 2 minute pulse 30 second pause group (p = 0.038). Time series traces of STAT5 ratio for non-responding cells are provided in Figure C in S1 File for comparison. Taken together, the results suggest that individual cell responses to repeated pulsatile IL-2 stimulus vary within Jurkat cell populations, and that the response profiles are affected by variation in pulse length and recovery time, with a stronger and faster response occurring at intermediate pulse times compared to both short and long pulse and recovery times.

## Discussion

Based on the reported ranges of subunit levels within T cell populations, including our previously reported results for gene expression of the IL-2 receptor subunits in primary T cells [21], we hypothesized that there would be population heterogeneity of cellular responses to IL-2 stimulation resulting from differences in the availability of unbound receptor subunits with which to form signal-inducing receptor-ligand complexes. In order to address this, we developed a model that enables us to simulate cell response with different levels of receptor subunits. In order to experimentally capture the variability in response at the single cell level and collect quantitative data, we employed a microfluidic device capable of trapping and arraying non-adherent cells for time series imaging. This allowed us to precisely replicate the modeled cytokine exposure on live cells and to longitudinally track protein translocation at the single cell level using live cell imaging. Our modeling approach aimed to investigate this by simulating cells with variable levels of individual receptor subunits. We reasoned that at below saturation levels of ligand, varying levels of receptor within a cell population would allow for differences in onset and duration of cellular response to ligand fluctuations in the extracellular environment. In order to test this, we introduced rapid fluctuations of IL-2 into our modeled system. A total of 36 combinations of time-dependent IL-2 stimulus were simulated, with pulse time and recovery pauses varied from 30 seconds to five minutes. Our model predicted that an input of repeated one minute IL-2 pulses should result in the comparatively broadest range of response profiles within a cell population based on initial IL-2Rα numbers per cell. Within our modeled system, internalization of receptor-ligand complex removes receptor subunits from the cell surface, and while IL-2Rα is recycled, IL-2Rβ and IL-2Rγ are not. Despite the production of new subunits, this makes the two latter subunits rate limiting in the cell’s response to IL-2. Our results pinpointed a one minute IL-2 pulse as the input range where this rate-limiting aspect of subunit availability had the greatest effect, a conclusion which was illustrated by the difference in maximum response between cells expressing high and low levels of IL-2Rβ and IL-2Rγ across input settings using a one minute pulse time. Likewise, a comparison of the shift in dynamic response for different input settings indicated that a one minute range should result in the widest range of intra-population response for Jurkat cells responding to 100 pM IL-2.

Variability of single-cell responses within populations has functional consequences, and the traditional approach of building computational models based on the average cell in a population fails to adequately capture this complex reality. Previous models of cellular interaction with IL-2 have simulated various aspects of interaction and downstream response, such as cell proliferation [26] or IL-2 induced cytokine production [45]. A common thread of the majority of studies has been the assumption that population averaged parameters and responses adequately represent biological reality. The growing awareness of the importance of a single cell approach has led to development of models focusing on aspects such as single cell competition for IL-2 which can have population level results on immune function [20]. While the Fallon & Lauffenburger model used as our starting point incorporated post-binding internalization and trafficking of the receptor and ligand, it focused on the commonly used downstream response of cell proliferation, which occurs on a time scale of days after IL-2 stimulation. In contrast, our model simulated an immediate response to IL-2, occurring within a time-frame of minutes, which can be expected to be unaffected by IL-2 induced gene upregulation. This allowed us to make the assumption that the individual cell response would be dependent on the initial state of the cell. Our model attempts to capture aspects of this variability within a cell population by allowing for variability of receptor subunit levels. The added detail of receptor subunits allowed a model description more closely adherent to the biological realities of differential subunit expression *in vivo*.

Our experimental microfluidic setup permitted investigation of single cell responses to IL-2 in a controlled setting, by simultaneously collecting response data from multiple cells within a population through the use of arrayed cell traps, orthogonal buffer flow, and longitudinal imaging. When subjected to a constant input of 100 pM IL-2, the cells in the population showed a range of responses, with one third of cells showing a clear translocation of GFP-labeled STAT5 from the cytosol to the nucleus over the one hour time course. This range of responses to identical stimulus highlights the importance of studying single cell response within populations, rather than relying on population averages, in order to fully understand the functionality of immune cell populations. The heterogeneity observed here suggests that varying levels of IL-2 receptor subunits at the single cell level can have functional consequences with regards to filtering dynamic IL-2 cues in the extracellular environment.

The initial resting ratios of nuclear to cytosolic STAT5-GFP in the cells indicated that STAT5 was present in the nucleus prior to experimental IL-2 stimulation, albeit at levels markedly lower than in the cytosol. This presence of nuclear STAT5 is presumably due to the effects of IL-2 exposure in culture, where Jurkat cells constitutively produce and release IL-2. This constitutive response to IL-2 results in a low level of downstream response with phosphorylation and translocation of cytosolic STAT5. Our population-averaged probing of both unlabeled and labeled STAT5 showed that the vast majority of STAT5 existed in the cytosolic space prior to IL-2 stimulation. We note also that based on our fluorescent images, the relative amount of STAT5-GFP in the nucleus varied between cells at resting state. No correlation was found between resting ratio of nuclear to cytosolic STAT5-GFP and the level of IL-2 induced translocation for individual cells. Cell-to-cell variability in signaling is commonly thought of as the result of accumulation of variation in protein at each level of a biochemical cascade. An alternate hypothesis to explain this variability states that rather than being caused by random variations in gene expression, it is caused by cellular convergence to specific attractor states within cell state space [46]. Such clustered heterogeneity could indicate the existence of distinct kinetic profiles within the cell population, allowing for the fulfillment of different functional needs.

The possibility that distinct cellular response states coexist in genetically identical cell populations prior to stimulation is an in intriguing addition to the discussion of cell-cell heterogeneity within immune cell populations. Our results showed distinct phenotypic subgroups within our cell population, with different response profiles to identical inputs. High responder cells showed initiation of quantifiable STAT5-GFP translocation soon after initial IL-2 pulse for the two intermediate pulse times of one and two minutes, indicating that these pulse times in combination with their respective recovery periods of one minute and 30 seconds provided a favorable input to fast cell response to extracellular IL-2. Interestingly, both shorter (30 seconds) and longer (five minutes) pulse and recovery times resulted in a slower initiation of STAT5-GFP translocation for high responding cells. This raises the possibility that the existence of different cell states within the population enables the cells to act as filters to initiate response within a preferred range of cytokine fluctuation while filtering out IL-2 input pulses that fall outside of this range.

The dynamics of receptor-ligand interaction in the IL-2 system, coupled with the relative speed of the downstream processes, suggests that this system could allow cells to distinguish between fluctuating pre-equilibrium (below steady state) levels of ligand [23]. Our microfluidic platform permitted testing of this prediction by precise control over extracellular IL-2 that let us investigate the effects of a pulsatile IL-2 input on the receptor-ligand complex dynamics in Jurkat cells. We exposed cells from the same population to IL-2 stimuli to combinations of stimulus pulse and recovery times, while simultaneously tracking downstream response for individual cells using fluorescent imaging of STAT5-GFP. The results provided a distribution of different response profiles within the population for all input settings. Interestingly, our results also showed differences in the onset of cell response and the spread of response strength as the pulsatile input was varied. Notably, short IL-2 pulses of 30 seconds followed by short 30 second recovery times showed a population profile similar to that seen when both IL-2 pulses and recovery times were long (five minutes). In contrast, intermediate pulse and pause times showed a faster onset of downstream response in the population, accompanied by a wider range of single cell response profiles. This indicates that Jurkat cells do indeed respond to pulsatile IL-2 input in a manner that is able to distinguish between pulsatile variations in extracellular IL-2 levels at below steady state concentrations. This is consistent with the prediction that the kinetics of the IL-2 receptor-ligand pair enables increased cellular sensitivity to fluctuating cytokine levels.

In an *in vivo* context, T cell response to IL-2 happens in a system subject to many control switches, such as *a priori* upregulation of IL-2Rα, and ligand competition both within the T cell population and between other cell types. By using a cell line which is pre-primed for IL-2Rα expression, we did not have to account for the need for IL-2Rα recruitment in order to initiate IL-2 response in the cells. In addition, our experimental setup allowed for precise control of IL-2 exposure while minimizing the number of biological control switches. Thus, our results indicate cell response to IL-2 stimulation in a context where the main contributors to cells response were IL-2 delivery and preexisting cell state of the responder cell.

Previously published data have reported a range of expression of IL-2 receptor subunits within T cell populations [20, 21]. While the expression levels of IL-2Rα are known to be affected by IL-2 due to priming of cells which initiates IL-2Rα upregulation, we used a cell line that is constitutively primed to express IL-2Rα. This allowed us to simulate how levels of other IL-2 receptor subunits (IL-2Rβ and IL-2Rγ) might alter cell responsiveness under primed conditions. In order to investigate the effects of initial cell to cell variability in receptor level, we modeled cells with high or low levels of these subunits and found that under primed conditions, the level of IL-2Rβ and IL-2γ became an indicator of cellular responsiveness to IL-2 as measured by downstream translocation of STAT5.

In conclusion, we have investigated single-cell responses to variable pulsatile IL-2 input, using a microfluidic platform in combination with time-lapse fluorescent imaging. Our results indicate that cell response profiles vary with varied pulsatile input at pre-equilibrium levels. By integrating computational tools with microfluidics, we were able to investigate both specified cell-cell variability and the spatiotemporal effects of controlled IL-2 stimulation. This combinatorial approach helped to increase our understanding of how cell-cell variability affects the range of cytokine responses within immune cell populations. We were limited in our goal of a single-cell approach by the scant availability of single cell information for the processes modeled, but by allowing for cell variability within ranges of subunit expression reported in literature, we captured the increase in population variance that resulted from the unique one minute on/off timescale. Our experimental setup allowed us to focus on cellular response at the single cell level, in a manner that complemented the computational model by highlighting the variability of the *in vitro* cell response within a population. An intriguing approach to incorporate single cell information into computational models by single cell parameter estimation was recently suggested by Yao et al [46]. By using Bayesian parameter inference at the single-cell level, followed by inferred parameter clustering, these investigators detected existing cellular states within their population, explaining previously observed response variability. Further development of our system using such an approach to incorporate our experimental information would allow for expanded interrogation of distinct cellular profiles. Furthermore, advances in longitudinal cytokine profiling of single immune cells on-chip [47] could be integrated with the platform applied here, allowing for information encoding and filtering of extracellular ligand levels to be related to functional heterogeneity of phenotype. The differences seen in maximum response and in response profiles with varying combinations of IL-2 pulse times and recovery times indicate that Jurkat cells have a preferred range of extracellular IL-2 fluctuations in which cellular response is initiated quickly, while cells are slower to respond and show lower response levels outside this fluctuation range. Further investigation into this filtering behavior could increase our understanding of how variability within immune cell populations enable a systems response within preferred fluctuation ranges and whether these ranges correspond to *in vivo* conditions.

## Supporting information

S1 File **Figure A.** Fitting delay function for STAT5 nuclear translocation. A logistic function was fitted to normalized experimental data from the mean of 21 responding cells (28% of population) stimulated with a constant input of 100 pM IL-2 over the course of one hour. Insets show STAT5-GFP in the same cell after 0, 30, and 60 minutes of IL-2 stimulation. **Figure B.** Relative amounts of STAT5 and STAT5-GFP in nucleus and cytosol. Western blot showing STAT5 and STAT5-GFP in the nuclear and cytosolic compartments of Jurkat cells prior to IL-2 stimulation. **Figure C.** Hierarchical clustering of live cell time series data was used to classify cells as responders or non-responders to IL-2. Clustered data for cells exposed to four input settings of periodic IL-2 stimulus: a) 30 seconds/30 seconds, b) one minute/one minute, c) two minutes/30 seconds, and d) five minutes/five minutes. **Figure D.** Heatmap showing the model predicted differences in AUC for receptor-ligand complexes per cell for cells with high vs low expression of IL-2Rβ/γ under 36 varying pulsatile IL-2 inputs. **Table A:** Model equations for all modeled species.
(DOCX)

S1 Movie STAT5 translocation in a Jurkat cell upon administation of bolus dose of 100 nM IL-2. Images taken at 60x every five minutes in the brightfield, GFP, and DAPI ranges.
(AVI)
