# Supplementary material for: Oscillatory IL-2 stimulus reveals pertinent signaling timescales of T cell responsiveness
Source: PLoS One. 2018 Sep 18;13(9):e0203759. doi: 10.1371/journal.pone.0203759 (PMC6143248; doi:10.1371/journal.pone.0203759)
Supplement: S1 File — Figure A. Fitting delay function for STAT5 nuclear translocation. A logistic function was fitted to normalized experimental data from the mean of 21 responding cells (28% of population) stimulated with a constant input of 100 pM IL-2 over the course of one hour. Insets show STAT5-GFP in the same cell after 0, 30, and 60 minutes of IL-2 stimulation. Figure B. Relative amounts of STAT5 and STAT5-GFP in nucleus and cytosol. Western blot showing STAT5 and STAT5-GFP in the nuclear and cytosolic compartments of Jurkat cells prior to IL-2 stimulation. Figure C. Hierarchical clustering of live cell time series data was used to classify cells as responders or non-responders to IL-2. Clustered data for cells exposed to four input settings of periodic IL-2 stimulus: a) 30 seconds/30 seconds, b) one minute/one minute, c) two minutes/30 seconds, and d) five minutes/five minutes. Figure D. Heatmap showing the model predicted differences in AUC for receptor-ligand complexes per cell for cells with high vs low expression of IL-2Rβ/γ under 36 varying pulsatile IL-2 inputs. Table A: Model equations for all modeled species. (DOCX) [file pone.0203759.s001.docx]

# Supporting Information

**
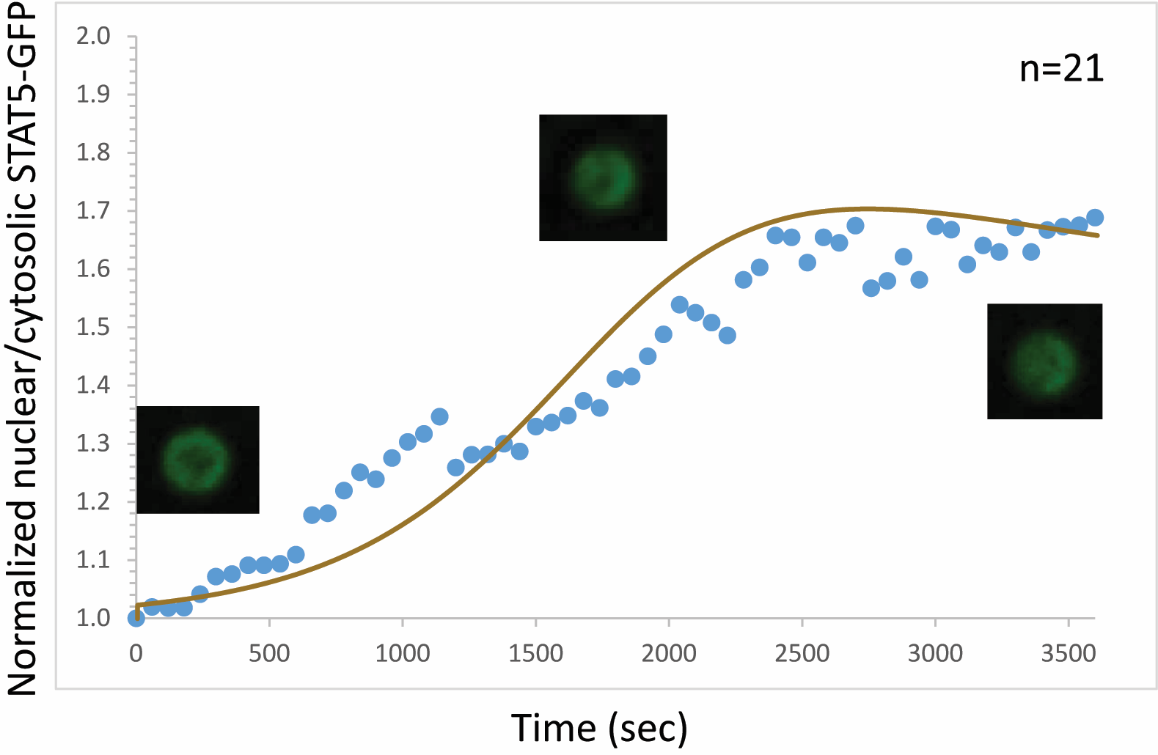
**

**Figure A.** Fitting delay function for STAT5 nuclear translocation. A logistic function was fitted to normalized experimental data from the mean of 21 responding cells (28% of population) stimulated with a constant input of 100 pM IL-2 over the course of one hour. Insets show STAT5-GFP in the same cell after 0, 30, and 60 minutes of IL-2 stimulation

**
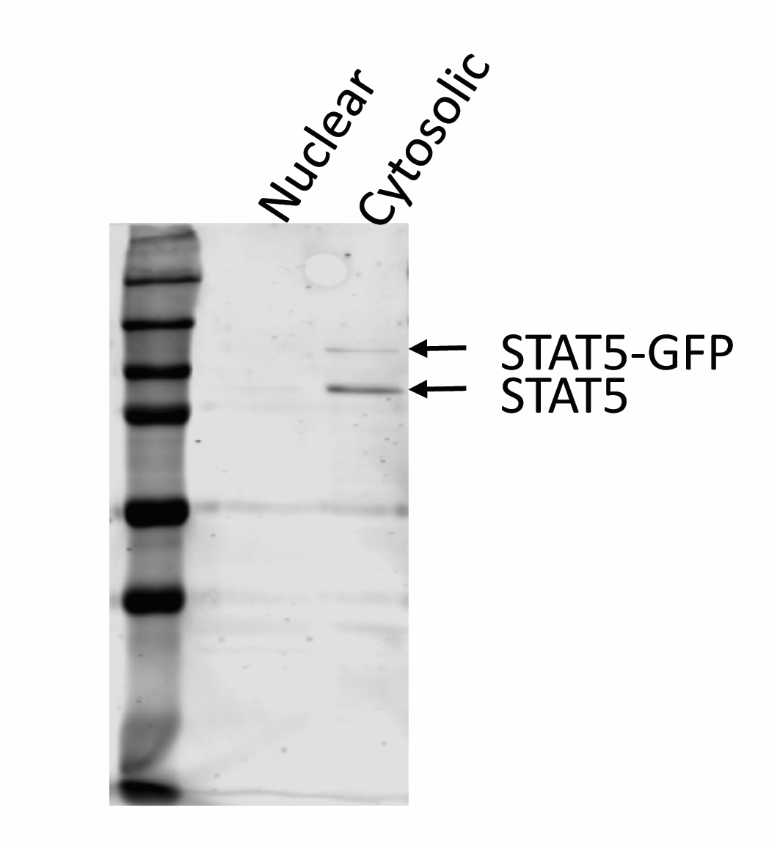
**

**Figure B.** Relative amounts of STAT5 and STAT5-GFP in nucleus and cytosol. Western blot showing STAT5 and STAT5-GFP in the nuclear and cytosolic compartments of Jurkat cells prior to IL-2 stimulation.

**
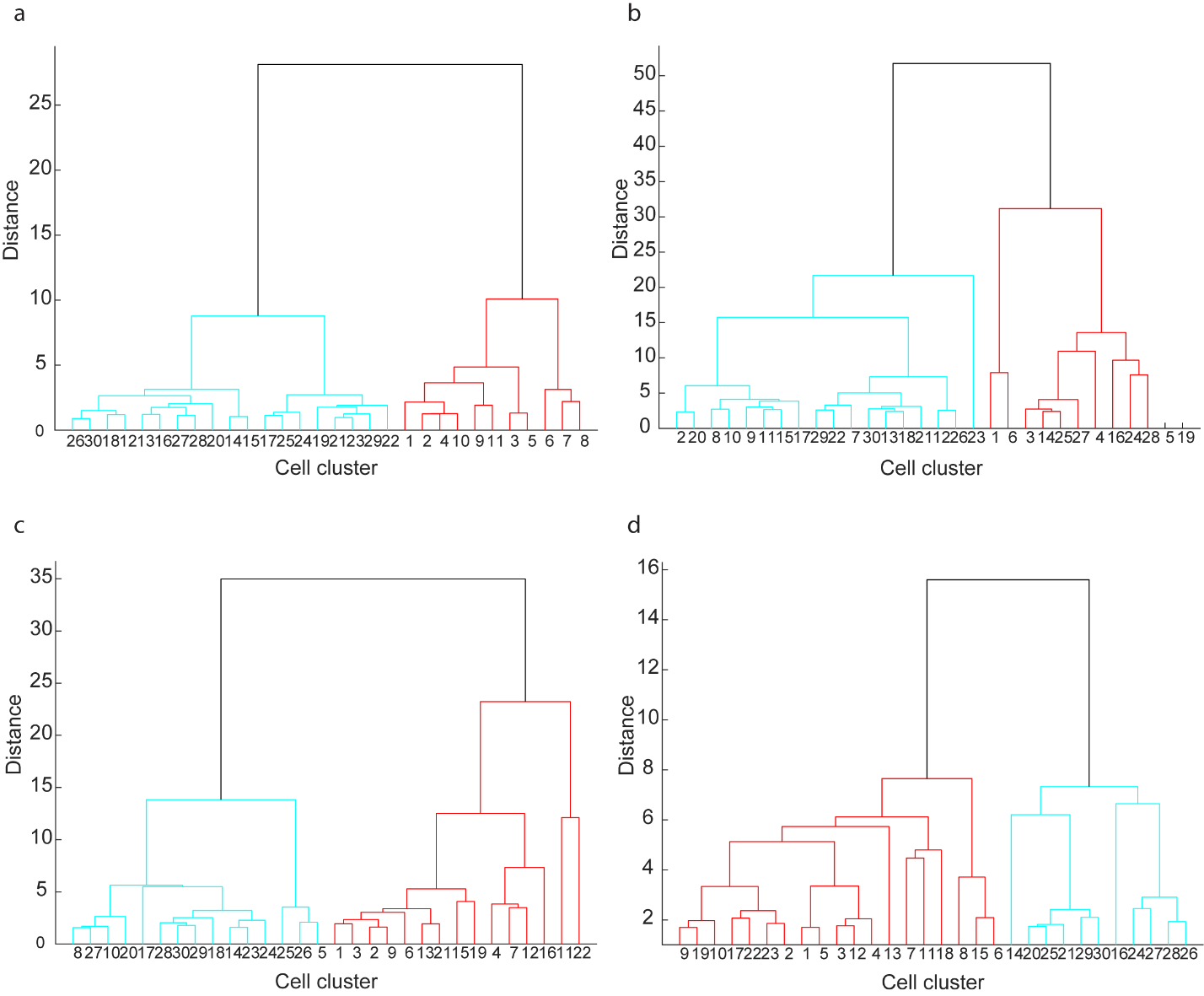
**

**Figure C.** Hierarchical clustering of live cell time series data was used to classify cells as responders or non-responders to IL-2. Clustered data for cells exposed to four input settings of periodic IL-2 stimulus: a) 30 seconds/30 seconds, b) one minute/one minute, c) two minutes/30 seconds, and d) five minutes/five minutes.

**
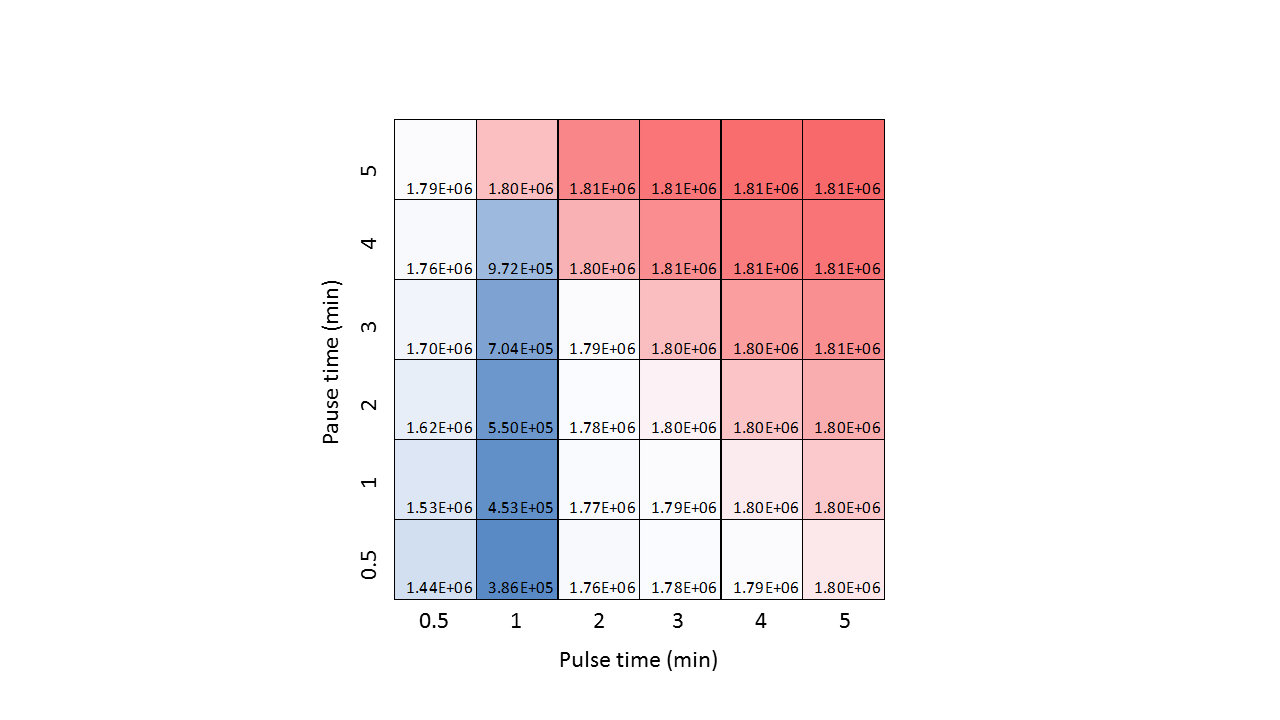
**

**Figure D.** Heatmap showing the model predicted differences in AUC for receptor-ligand complexes per cell for cells with high vs low expression of IL-2Rβ/γ under 36 varying pulsatile IL-2 inputs.

**Table A:** Model equations for all modeled species.

| **Species** | **Equation** |
| --- | --- |
| Cell surface receptor-ligand complex | dCs/dt = 1/((kon2*LA*BG)-((kr*Cs)+(ke*Cs)) |
| Intracellular receptor-ligand complex | dRi/dt = 1/((kt*Rs)+(kre*Ci))-((kfe*Li*Ri)+(kh*Ri)) |
| Intracellular ligand | dLi/dt = 1/((kre*Ci)/(Ve*NA))-((kfe*Li*Ri)/(Ve*NA)+(kx*Li)) |
| Intracellular receptor-ligand complex | dCi/dt = 1/((kfe*Li*Ri)+(ke*Cs))-((kre+kh*Ci)) |
| Cell surface IL-2Rα | dA/dt = 1/((koff1*LA)+(ksyn*Cs)+Vs)-(kon1*L*A) |
| Cell surface IL-2Rβγ | dBG/dt = 1/((kr*Cs*Vs)-(kon2*LA*BG)) |
| Cell surface IL-2Rα-ligand complex | dLA/dt = 1/((kon1*L*A)+(kr*Cs))-((koff1*LA)+(kon2*LA*BG)) |
| Delay function for STAT5 translocation | Sr=y+(b*Cs)/(d+exp(-ku1*(t-ti))) |
